# Virus-Dependent Relative Contributions of Citric Acid and Benzalkonium Chloride to the Virucidal Efficacy of Combination Disinfectants

**DOI:** 10.3390/microorganisms14091873

**Published:** 2026-08-24

**Authors:** Sok Song, Kyu-Sik Shin, So-Hee Park, Yong Yi Joo, Cho-Yeon Lee, Hyun-Ok Ku, Wooseog Jeong

**Affiliations:** Veterinary Drugs & Biologics Division, Animal and Plant Quarantine Agency, Gimcheon 39660, Gyeongbuk-do, Republic of Korea; ssoboro@korea.kr (S.S.); soso9354@naver.com (K.-S.S.); sh0526@korea.kr (S.-H.P.); wndyddl7091@korea.kr (Y.Y.J.); sol77lee@korea.kr (C.-Y.L.); kuho@korea.kr (H.-O.K.)

**Keywords:** combination disinfectants, virucidal efficacy, FMDV, AIV, Citric acid, benzalkonium chloride

## Abstract

Chemical disinfectants used in livestock production commonly combine citric acid (CA) and benzalkonium chloride (BZK), yet the respective contributions of these active ingredients to virucidal efficacy remain poorly understood. This study quantified the relative contributions of CA and BZK against the non-enveloped foot-and-mouth disease virus (FMDV) and the enveloped avian influenza virus (AIV). Virucidal efficacy was evaluated using a full-factorial design comprising six CA concentrations and six BZK concentrations at contact times of 3 and 30 min. Log reduction values (LRVs) were analyzed by two-way ANOVA and multiple linear regression, and the relative importance of each predictor was estimated using the Lindeman–Merenda–Gold (LMG) method. FMDV inactivation was primarily determined by CA, which accounted for 99.99% and 99.87% of the explained variance at 3 and 30 min, respectively, whereas BZK and the interaction term contributed minimally. In contrast, BZK was the dominant determinant of AIV inactivation, explaining 69.79% and 78.02% of the variance at 3 and 30 min, respectively, while CA made a smaller contribution. These findings demonstrate that the dominant active ingredient in CA–BZK combination disinfectants differed markedly between the FMDV and AIV models examined. Quantifying the relative contribution of individual components provides a basis for the rational formulation and optimization of veterinary disinfectants for different target viruses.

## 1. Introduction

Foot-and-mouth disease (FMD) and highly pathogenic avian influenza (HPAI) are among the most devastating transboundary animal diseases, causing substantial economic losses to the livestock industry and international trade worldwide [1,2,3,4,5]. Because both diseases can spread not only through infected animals but also indirectly via contaminated environments, vehicles, equipment, and personnel, rapid elimination of environmental pathogens is a critical component of outbreak control, and chemical disinfection is widely implemented as a primary biosecurity measure [2,6]. Consequently, the selection and optimization of disinfectant formulations have become increasingly important for improving farm biosecurity and minimizing the risk of indirect disease transmission [7,8].

Despite their similar epidemiological importance, foot-and-mouth disease virus (FMDV) and avian influenza virus (AIV) differ fundamentally in their structural characteristics. FMDV, a non-enveloped RNA virus belonging to the Picornaviridae family, lacks a lipid membrane, whereas AIV is an enveloped RNA virus possessing a lipid envelope [9,10,11]. This structural difference is widely recognized as a key determinant of susceptibility to chemical disinfectants, often leading to substantial variation in virucidal efficacy depending on the target virus [9,12,13,14]. Organic acids, particularly citric acid, primarily inactivate viruses by lowering environmental pH, thereby destabilizing acid-sensitive viral proteins or capsid structures [15,16,17]. In contrast, quaternary ammonium compounds (QACs), including benzalkonium chloride, exert their virucidal activity mainly through interactions with lipid membranes, resulting in disruption of the viral envelope [18,19,20]. Consequently, organic acids and QACs are expected to exhibit distinct efficacy profiles depending on the structural and physicochemical properties of the target virus.

Our previous study evaluated four disinfectant active ingredients individually across four transboundary animal disease viruses and characterized their concentration- and time-dependent efficacy using single-component response profiles and critical concentrations [21]. Within that framework, FMDV showed high susceptibility to CA and limited susceptibility to BZK, whereas AIV showed greater susceptibility to BZK than to CA. These observations established ingredient-specific susceptibility patterns but did not address the behavior of the two components when they coexist in a formulation. In particular, the previous design could not determine whether virucidal efficacy within a CA–BZK mixture is predominantly attributable to one component, is shared between the two components, or is influenced by their interaction. To address this distinct question, the present study employed a 6 × 6 full-factorial CA–BZK matrix in which both component concentrations were varied simultaneously and independently. This design allowed the effects of CA, BZK, and their interaction within the combination system to be evaluated simultaneously and their relative contributions to the variation in virucidal efficacy to be quantitatively assessed for FMDV and AIV.

However, many commercially available disinfectants used in livestock production contain both CA and BZK as combined active ingredients, representing one of the most widely used disinfectant formulations in veterinary practice. The rationale for combining different classes of disinfectants is generally to broaden antimicrobial spectrum and improve overall efficacy under diverse field conditions [22,23]. Nevertheless, the relative contribution of each active ingredient within these formulations is rarely evaluated experimentally, and efficacy is generally assessed only for the final commercial product. As a result, it remains unclear whether both components actively contribute to virucidal activity or whether one ingredient predominantly determines the observed efficacy. Despite the widespread use of such combination formulations, the respective contributions of each ingredient within these mixtures remain unclear. In particular, it is unknown whether the observed efficacy is driven by a single dominant component or whether the relative contribution of each ingredient varies depending on the target virus. This gap is especially relevant given the opposing activity profiles of CA and BZK observed in FMDV and AIV [21].

Therefore, the present study applied an identical concentration-combination experimental design to both non-enveloped FMDV and enveloped AIV to systematically investigate the virucidal behavior of CA–BZK formulations. A full factorial concentration matrix was employed to independently vary the concentrations of both active ingredients over a broad experimental range, enabling quantitative assessment of their individual effects as well as their combined influence on virucidal efficacy. By quantitatively comparing viral inactivation across a wide range of concentration combinations, we aimed to determine the relative contribution of each active ingredient and to characterize their interaction patterns in each virus. Ultimately, this study provides a quantitative basis for understanding how the functional contributions of disinfectant ingredients differ between the FMDV and AIV models examined and offers a scientific framework for evaluating combination disinfectants used in livestock disease control.

## 2. Materials and Methods

### 2.1. Viruses and Cell Lines

Foot-and-mouth disease virus (FMDV; serotype O, vaccine strain O/SKR/Boeun/2017; KVCC-VR1700004) and avian influenza virus (AIV; H9N2 strain A/chicken/Korea/MS96/1996; KVCC-VR1100013) were used as representative non-enveloped and enveloped viruses, respectively. All virus stocks were obtained from the Korea Veterinary Culture Collection (KVCC, Gimcheon, Republic of Korea) and handled in biosafety level (BSL)-appropriate facilities at the Animal and Plant Quarantine Agency (APQA). FMDV was propagated in LFBK cells (porcine fetal kidney-derived; KVCC, Gimcheon, Republic of Korea), whereas AIV was propagated in Madin–Darby canine kidney (MDCK) cells (ATCC^®^ CCL-34™, Manassas, VA, USA).

Cells were maintained in Dulbecco’s Modified Eagle’s Medium (DMEM) or Minimum Essential Medium (MEM) (Corning Inc., Corning, NY, USA) supplemented with 5–10% fetal bovine serum (FBS), Antibiotic–Antimycotic solution (Gibco, Thermo Fisher Scientific, Waltham, MA, USA), and 200 mM L-glutamine (Gibco, Thermo Fisher Scientific, Waltham, MA, USA), and were incubated at 37 °C in a humidified atmosphere containing 5% CO_2_.

### 2.2. Disinfectant Preparation and Experimental Design

**Figure 1:**
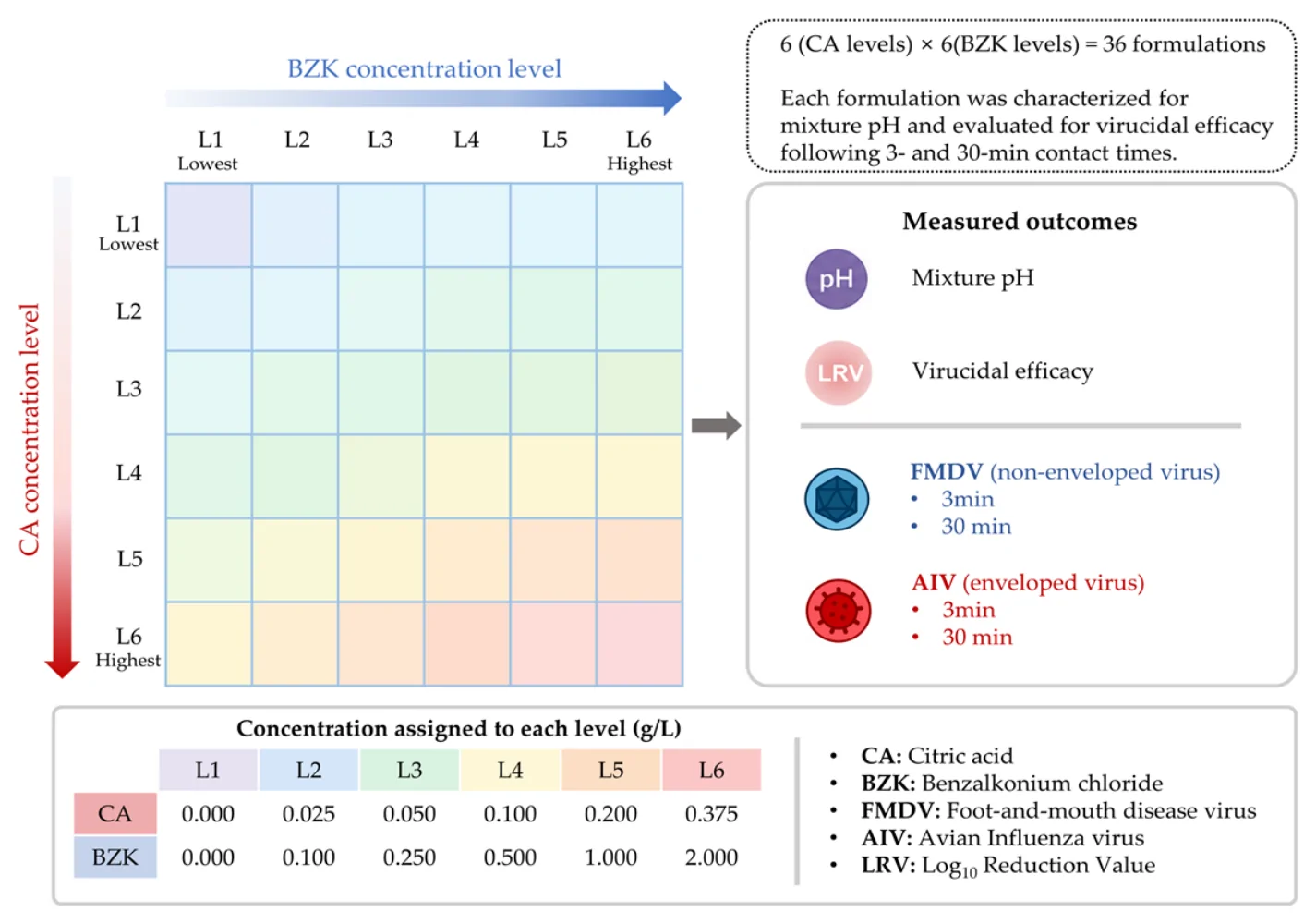
Experimental design of the CA–BZK full-factorial study. Six concentration levels of citric acid (CA) and benzalkonium chloride (BZK) were combined to generate 36 formulations. Each formulation was evaluated for mixture pH and virucidal efficacy against FMDV and AIV after 3- and 30-min contact times.

Citric acid (CA; ≥99.5%, ACS reagent grade; Cat. No. 251275) and benzalkonium chloride (BZK; ≥50% via Cl, 50% in H_2_O; Cat. No. 63249) were selected as representative organic acid and quaternary ammonium compound disinfectants and were purchased from Sigma–Aldrich (St. Louis, MO, USA). A full factorial experimental design consisting of six CA concentration levels and six BZK concentration levels (6 × 6) was employed, resulting in a total of 36 unique formulations (Figure 1).

CA concentrations were set at 0.000, 0.025, 0.050, 0.100, 0.200, and 0.375 g/L, while BZK concentrations were set at 0.000, 0.100, 0.250, 0.500, 1.000, and 2.000 g/L. These concentration ranges were selected based on our previous single-component study together with preliminary range-finding experiments, ensuring inclusion of concentrations spanning ineffective, partially effective, and fully effective virucidal responses [21].

All disinfectant solutions were freshly prepared using standard hard water (300 ppm as CaCO_3_) immediately prior to testing and were used within 24 h. Mixed formulations were prepared by combining CA and BZK at the designated concentrations for each experimental condition.

The pH of each formulation was measured immediately after preparation at room temperature using a calibrated SevenCompact™ pH meter S220 (Mettler Toledo, Greifensee, Switzerland). The measured pH values were recorded for all formulations to evaluate the contribution of solution acidity to virucidal efficacy.

### 2.3. Virucidal Efficacy Assay

Virucidal efficacy was evaluated using a quantitative suspension assay in accordance with APQA guidelines. For each experimental condition, 500 µL of disinfectant solution was mixed with 500 µL of virus suspension at a 1:1 ratio to obtain a total reaction volume of 1 mL, and the mixture was incubated at 4 °C for the designated contact times of 3 min and 30 min. Immediately after the contact period, disinfectant activity was neutralized by adding an equal volume of cell culture medium supplemented with 10% fetal bovine serum (FBS), and the efficiency of neutralization and the absence of residual disinfectant activity were verified using control recovery assays. The neutralized samples were subsequently subjected to tenfold serial dilution (10^0^–10^−6^), and aliquots were inoculated onto appropriate host cells (LFBK for FMDV and MDCK for AIV) in 96-well plates. Inoculated cells were incubated at 37 °C in a humidified atmosphere containing 5% CO_2_ for 5–7 days and examined daily by light microscopy for virus-induced cytopathic effects (CPE). CPE was determined by the presence of characteristic morphological changes in inoculated cell monolayers relative to uninfected cell controls, and each well was classified as CPE-positive or CPE-negative. The proportion of CPE-positive wells at each dilution was used to determine the endpoint virus titer, which was calculated using the Spearman–Kärber method [24]. Virucidal efficacy was expressed as the log_10_ reduction value (LRV), calculated as the difference between the mean viral titer of untreated controls and that of disinfectant-treated samples. A reduction of ≥ 4 log_10_ (≥99.99% inactivation) was considered indicative of effective virucidal efficacy according to internationally accepted veterinary disinfectant efficacy standards [25,26,27]. To distinguish disinfectant-induced cytotoxicity from virus-induced CPE, parallel cytotoxicity controls were included in which disinfectant solutions were applied to cells in the absence of virus.

### 2.4. Data Analysis and Statistical Evaluation

All experiments were performed in triplicate (*n* = 3), and virucidal efficacy was expressed as the mean log_10_ reduction value (LRV) ± standard deviation (SD). Statistical analysis was performed using two-way ANOVA followed by Tukey’s multiple comparison test, with *p* < 0.05 considered statistically significant.

To evaluate the relative contributions of CA and BZK, multiple linear regression analyses were performed separately for each virus and contact time using CA concentration, BZK concentration, and their interaction (CA × BZK) as predictor variables. Standardized regression coefficients (*β*) and relative importance were estimated using the Lindeman–Merenda–Gold (LMG) method, and relative contributions were expressed as percentages of the explained variance (R^2^).

Heatmaps, interaction plots, forest plots, and relative contribution plots were generated to visualize the data. Statistical analyses and graphical visualizations were performed using GraphPad Prism (version 10.5.0; GraphPad Software, San Diego, CA, USA), Microsoft Excel 2021 (Microsoft Corporation, Redmond, WA, USA), and Python (version 3.11).

## 3. Results

### 3.1. Mixture pH of CA–BZK Formulations

**Figure 2:**
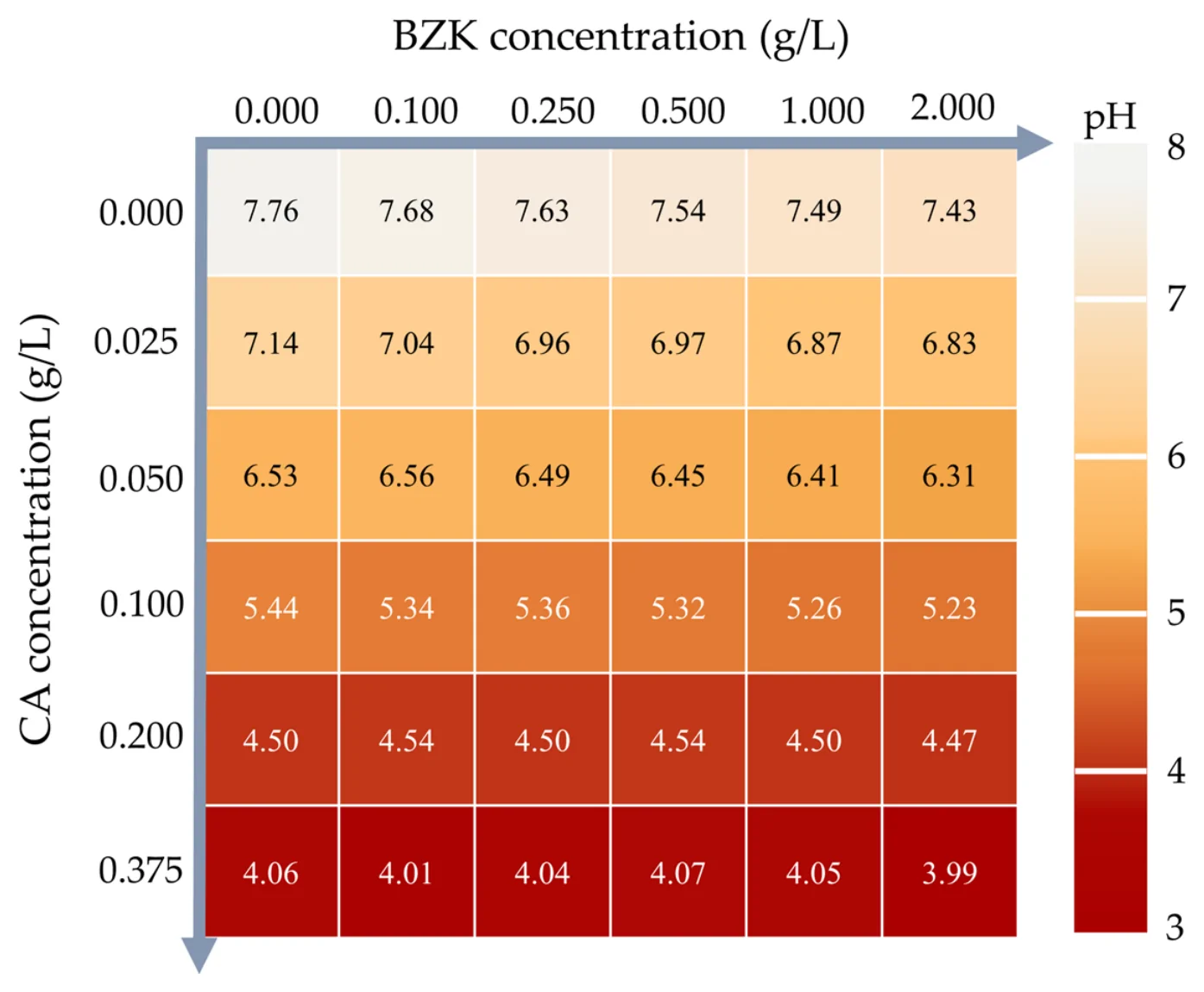
Mixture pH of CA–BZK formulations. Mixture pH values of the 36 CA–BZK formulations arranged according to CA and BZK concentrations.

The pH values of all 36 CA–BZK formulations are presented in Figure 2. Mixture pH decreased progressively with increasing CA concentration, ranging from 7.76 in the formulation without CA or BZK to 3.99 in the formulation containing the highest concentrations of both CA (0.375 g/L) and BZK (2.000 g/L).

Across each CA concentration level, increasing BZK concentration produced only minor changes in pH, with differences generally less than 0.3 pH units.

In contrast, variation in CA concentration was the primary determinant of mixture pH across the entire experimental range.

These findings indicate that the acidity of the mixed formulations was primarily determined by CA concentration, whereas BZK contributed minimally to the overall pH. Therefore, any subsequent differences in virucidal efficacy associated with increasing BZK concentration are unlikely to be attributable to changes in solution acidity.

### 3.2. Virucidal Efficacy of CA–BZK Formulations Against FMDV

**Figure 3:**
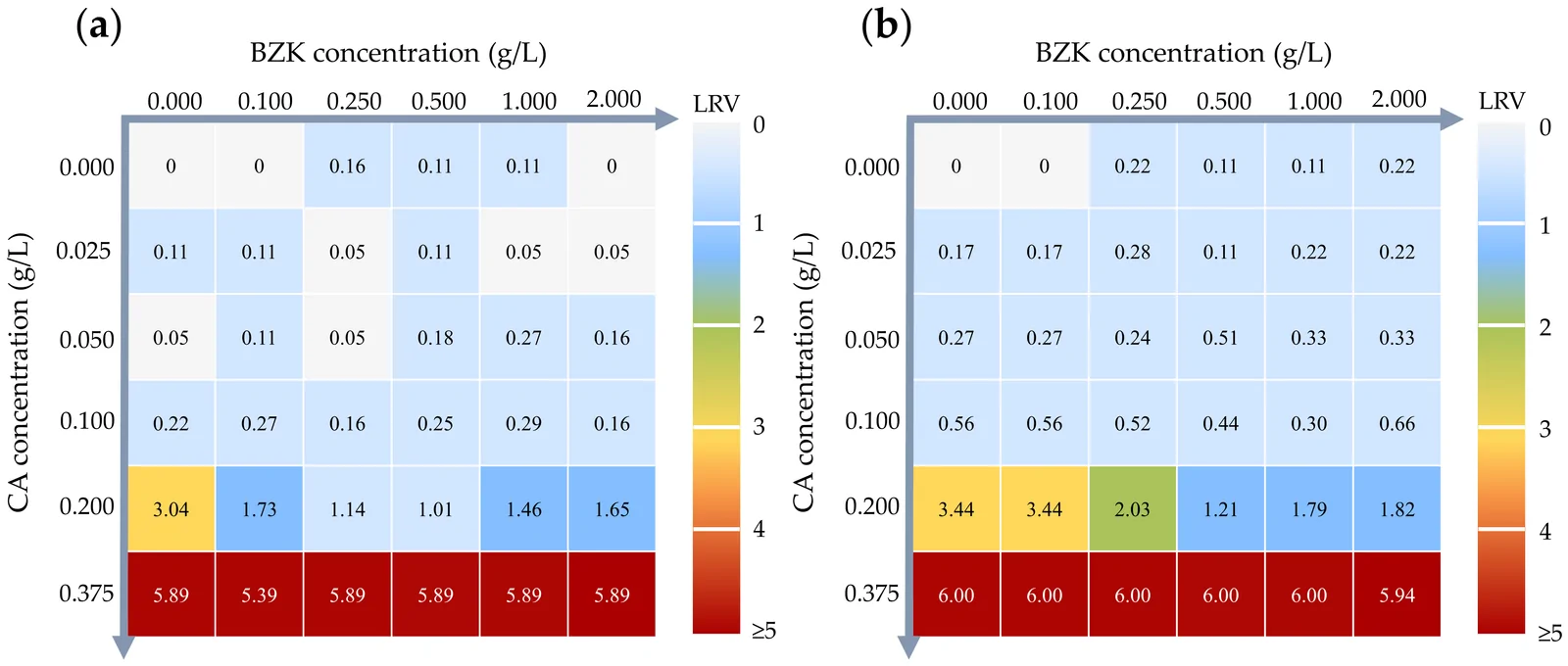
Virucidal efficacy of CA–BZK formulations against FMDV. Heatmaps of LRVs for the 36 CA–BZK formulations following (**a**) 3- and (**b**) 30-min contact times. Each cell represents the mean LRV of three independent experiments (n = 3). Values ≥ 5 indicate complete virus inactivation within the detection limit of the assay. Color intensity represents increasing virucidal efficacy.

The virucidal efficacy of the 36 CA–BZK formulations against FMDV after 3- and 30-min contact is summarized in Figure 3. Each value shown in the heatmaps represents the mean LRV obtained from three independent replicates. Control recovery assays performed to assess the neutralization procedure yielded recoverable FMDV titers ranging from 5.83 to 6.17 log_10_ TCID_50_/mL following dilution with culture medium supplemented with 10% FBS.

After 3 min of contact (Figure 3a), formulations containing 0.000–0.100 g/L CA exhibited minimal virucidal activity regardless of BZK concentration, with mean LRVs generally remaining below 0.3. Increasing the CA concentration to 0.200 g/L markedly increased antiviral activity, yielding mean LRVs ranging from 1.01 to 3.04 depending on the BZK concentration. At the highest CA concentration (0.375 g/L), all formulations achieved mean LRVs of 5.39–5.89, corresponding to complete virus inactivation irrespective of the BZK concentration.

A similar concentration-dependent pattern was observed after 30 min of contact (Figure 3b). Formulations containing 0.000–0.100 g/L CA remained largely ineffective (mean LRVs ≤ 0.66), whereas 0.200 g/L CA produced substantially greater reductions, with mean LRVs ranging from 1.21 to 3.44. All formulations containing 0.375 g/L CA achieved complete inactivation, with mean LRVs of 5.94–6.00.

Across both contact times, increasing the BZK concentration from 0.000 to 2.000 g/L produced only limited changes in virucidal efficacy within each CA concentration level, whereas increasing CA concentration resulted in a pronounced increase in FMDV inactivation. These findings indicate that increasing CA concentration exerted a substantially greater influence on FMDV inactivation than increasing BZK concentration.

### 3.3. Virucidal Efficacy of CA–BZK Formulations Against AIV

**Figure 4:**
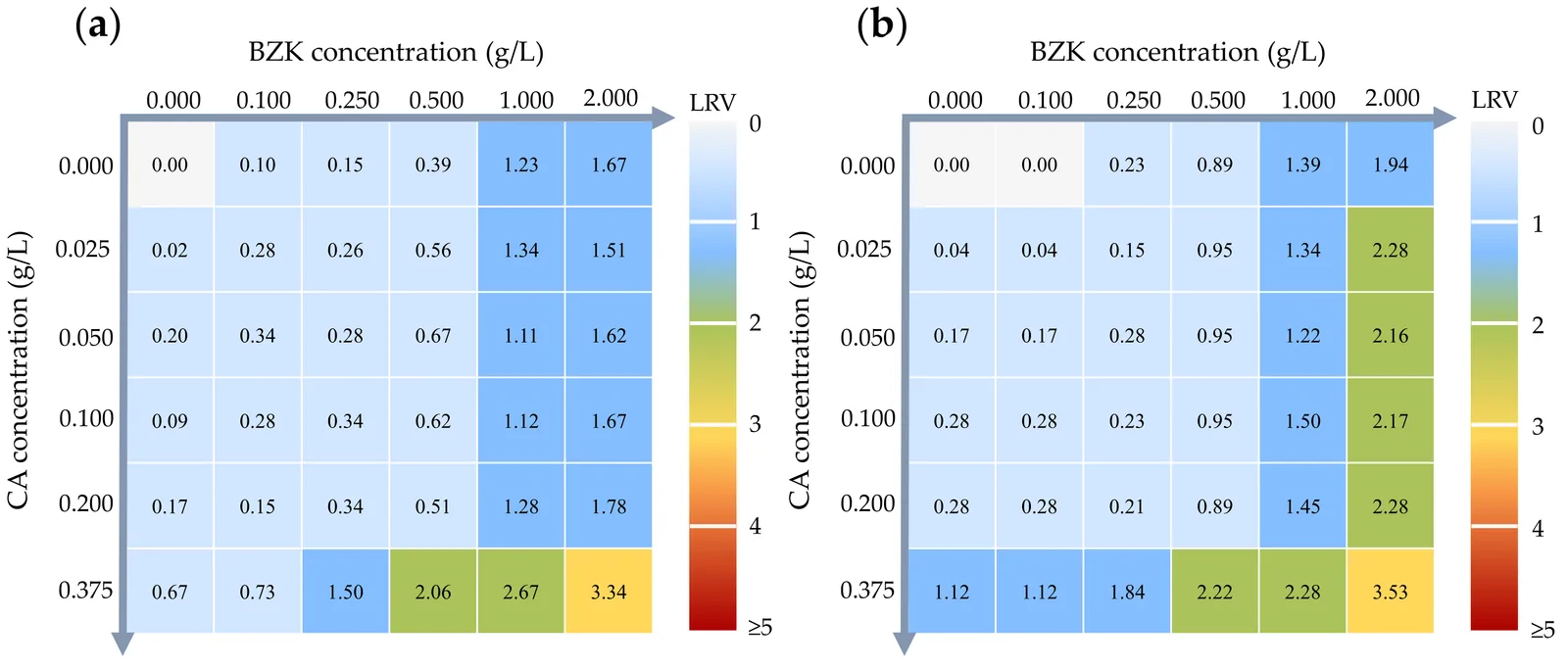
Virucidal efficacy of CA–BZK formulations against AIV. Heatmaps of LRVs for the 36 CA–BZK formulations following (**a**) 3- and (**b**) 30-min contact times. Each cell represents the mean LRV of three independent experiments (n = 3). Values ≥ 5 indicate complete virus inactivation within the detection limit of the assay. Color intensity represents increasing virucidal efficacy.

Figure 4 shows the virucidal efficacy of the 36 CA–BZK formulations against AIV after 3 and 30 min of contact. Control recovery assays performed to assess the neutralization procedure yielded recoverable AIV titers ranging from 6.17 to 6.33 log_10_ TCID_50_/mL following dilution with culture medium supplemented with 10% FBS. Overall, virucidal activity increased predominantly with increasing BZK concentration, whereas variation in CA concentration produced comparatively small changes in LRV.

After 3 min of contact (Figure 4a), formulations containing 0–0.5 g/L BZK generally produced LRVs below 1.0 across all CA concentrations. Increasing the BZK concentration to 1.0 and 2.0 g/L resulted in a marked increase in virucidal efficacy, with LRVs reaching 1.23–2.67 and 1.51–3.34, respectively. Increasing the CA concentration alone produced only modest improvements in LRV, although the highest LRVs were observed when the highest concentrations of both CA (0.375 g/L) and BZK (2.0 g/L) were combined.

A similar concentration-dependent pattern was observed after 30 min of contact (Figure 4b). Prolonged exposure increased virucidal efficacy across the entire concentration matrix, particularly at BZK concentrations of 1.0 and 2.0 g/L. The maximum LRV was 3.53 for the formulation containing 0.375 g/L CA and 2.0 g/L BZK. In contrast, formulations containing low BZK concentrations continued to exhibit limited antiviral activity regardless of CA concentration.

Overall, the heatmaps demonstrate that BZK concentration was the primary factor associated with increased virucidal efficacy against AIV, whereas CA concentration had comparatively little effect.

### 3.4. Quantitative Partitioning of CA and BZK Contributions Within Combination Formulations

**Figure 5:**
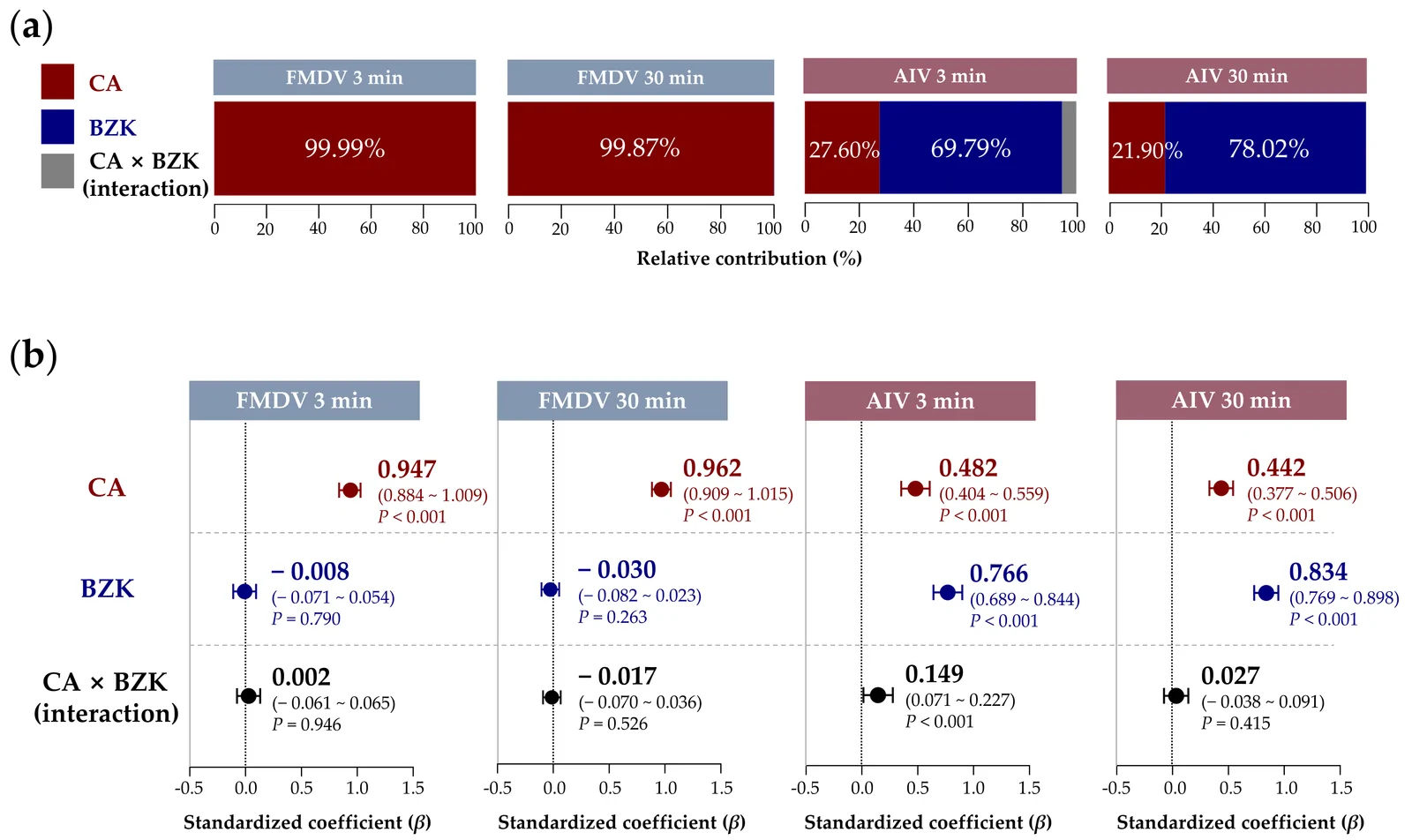
Quantitative partitioning of component contributions to virucidal efficacy within CA–BZK combination formulations. (**a**) Relative contributions of CA, BZK, and the CA × BZK interaction to the explained variation in LRV, estimated using the LMG method separately for each virus and contact time. Percentages represent each predictor’s share of the total explained variance within the full-factorial formulation matrix. (**b**) Standardized regression coefficients (*β*) with 95% confidence intervals from the corresponding multiple linear regression models. *p* values are shown for each predictor.

The heatmaps in Figure 3 and Figure 4 describe how virucidal efficacy changes across individual CA–BZK concentration combinations, but they do not quantify the relative contribution of each component across the factorial design. We therefore applied multiple regression and LMG relative-importance analysis to partition the explained variance in LRV among CA concentration, BZK concentration, and the CA × BZK interaction separately for each virus and contact time (Figure 5). This analysis addresses a different question from the single-component efficacy comparisons in our previous study [21]: it quantifies the relative explanatory importance of each component when both are varied within the same formulation matrix.

For FMDV, CA accounted for virtually all explained variance in virucidal efficacy (99.99% at 3 min and 99.87% at 30 min), whereas the contributions of BZK and the interaction term were negligible (<0.1%). In contrast, BZK was the predominant contributor to AIV inactivation, explaining 69.79% and 78.02% of the variance at 3 and 30 min, respectively, while CA accounted for 27.60% and 21.90%. The interaction term contributed only minimally (<3%).

Standardized regression coefficients were consistent with the relative importance analysis (Figure 5b). CA was the only significant predictor of FMDV inactivation (*β* = 0.947 and 0.962 at 3 and 30 min, respectively; both *p* < 0.001), whereas BZK showed no significant effect. Conversely, both CA and BZK significantly influenced AIV inactivation, although BZK exhibited substantially larger standardized coefficients (*β* = 0.766 and 0.834) than CA (*β* = 0.482 and 0.442). The interaction term showed only a small effect and was significant only at 3 min.

Together, these analyses quantitatively demonstrate that virucidal efficacy of CA–BZK formulations is governed primarily by CA against FMDV but by BZK against AIV.

## 4. Discussion

The present study systematically quantified the relative contributions of CA, BZK, and their interaction to the virucidal efficacy of combination disinfectants against representative non-enveloped and enveloped animal viruses. Commercial veterinary disinfectants frequently contain multiple active ingredients, yet the respective contribution of each component has rarely been quantified [28,29,30,31]. Using an identical full-factorial experimental design for FMDV and AIV, the present study demonstrated that the dominant active ingredient differed markedly between the two virus models examined. CA accounted for virtually all explained variance in FMDV inactivation, whereas BZK was the principal determinant of AIV virucidal efficacy. The interaction between the two components contributed minimally, indicating that virucidal efficacy was primarily determined by the dominant active ingredient rather than by synergistic effects.

The predominance of CA against FMDV is consistent with the well-established acid sensitivity of aphthoviruses [16,32,33]. Exposure to acidic conditions causes irreversible dissociation of the FMDV capsid, resulting in loss of infectivity [34,35,36]. Consistent with this mechanism, increasing CA concentration markedly reduced the pH of the disinfectant mixtures and produced corresponding increases in virucidal efficacy, whereas increasing BZK concentration had little effect on pH or virus inactivation. These findings are consistent with acidification being the primary mechanism governing FMDV inactivation under the experimental conditions examined and help explain the negligible contribution of BZK observed in the present formulation matrix.

In contrast, AIV exhibited a markedly different response pattern. BZK accounted for approximately 70–78% of the explained variance, whereas CA made a comparatively smaller contribution. This observation is consistent with the established mechanism of quaternary ammonium compounds, which act mainly through disruption of the viral lipid envelope [18,19,37,38]. Although increasing CA concentration produced modest improvements in virucidal efficacy, these effects remained secondary to those of BZK, suggesting that membrane-active effects associated with BZK played a greater role than acidification in determining AIV susceptibility within the concentration range evaluated.

The relationship between the present findings and our previous study [21] warrants explicit distinction. Reference [21] addressed single-component virucidal susceptibility by evaluating CA, BZK, and other active ingredients separately across concentration and contact-time gradients. That study therefore established which individual ingredients were relatively effective against each virus but did not estimate their relative functional contributions when two ingredients were present simultaneously. The present study deliberately narrowed the experimental scope to CA, BZK, FMDV, and AIV in order to address this second question using a 6 × 6 full-factorial combination matrix.

The resulting LMG analysis provided information that cannot be derived directly from the previous single-component potency profiles. In FMDV, 99.99% and 99.87% of the explained variance in mixture efficacy was attributed to CA at 3 and 30 min, respectively, whereas BZK and the fitted interaction term contributed negligibly. In AIV, by contrast, the explained variance was distributed primarily to BZK (69.79% and 78.02%), with smaller but measurable contributions from CA (27.60% and 21.90%). The interaction term contributed less than 3%. Accordingly, the interaction itself is not the sole novel outcome of the present study; rather, the main advance is quantitative partitioning of the relative contributions of both active ingredients and their interaction within a combination formulation.

These findings have practical implications for the development and evaluation of veterinary disinfectants. The results indicate that the contribution of each active ingredient depends strongly on the target virus. For the FMDV model examined here, formulation optimization would primarily require sufficient acidification, whereas for the AIV model, maintaining adequate concentrations of membrane-active compounds such as BZK is likely to have a greater impact on efficacy. More broadly, evaluation of combination disinfectants should consider not only overall virucidal efficacy but also the functional contribution of individual active ingredients, thereby providing a more rational basis for formulation design.

Several limitations should be acknowledged. First, the study included one FMDV strain and one AIV strain; therefore, the observed contribution patterns should not be generalized to non-enveloped and enveloped viruses as broad categories. Rather, the present results demonstrate marked differences between the two virus models examined. Second, the study investigated only one organic acid–quaternary ammonium compound combination, CA–BZK, and the relative contributions observed here may differ with other active ingredients or formulation systems. Finally, the interaction analysis was based on statistical modeling and did not directly investigate the molecular mechanisms underlying virus inactivation. Nevertheless, the factorial approach used here provides a framework for quantitatively evaluating component-level contributions in other combination disinfectants. Future studies incorporating additional virus species, disinfectant classes, and mechanistic analyses will be needed to determine the broader applicability of these findings.

## 5. Conclusions

This study quantified the component-specific contributions of CA and BZK within full-factorial combination formulations, extending our previous single-component efficacy observations. CA accounted for 99.99% and 99.87% of the explained variance in FMDV inactivation at 3 and 30 min, respectively, whereas BZK accounted for 69.79% and 78.02% of the explained variance in AIV inactivation, with CA contributing 27.60% and 21.90%. The CA × BZK interaction contributed only minimally under the tested conditions. These results demonstrate virus-dependent allocation of virucidal efficacy within the CA–BZK formulations examined and provide a quantitative framework for evaluating the functional contribution of individual components in combination disinfectants.

## Data Availability

The data supporting the findings of this study are available within the article.

## Abbreviations

The following abbreviations are used in this manuscript:

AIV	Avian influenza virus
BZK	Benzalkonium chloride
CA	Citric acid
FMDV	Foot-and-mouth disease virus
LRVs	Log reduction values
QACs	Quaternary ammonium compounds
